# Monthly Summary

**Published:** 1899-12

**Authors:** 


### Monthly Summary.

UNDER ONE FLAG.—One of the most conducive evils checking the united progress of dental education in this country is the innumerable degrees conferred on our dental graduates. The mother profession (medicine) is surprised to know that such is the case.

Medicine, with all its years of existence, has had but one degree, M. D., the world over. For the past fifteen years I have studied the evils of these innumerable degrees. I have taken up the subject and have solved it as one would solve a problem in mathematics. I have arrived at the full conclusion that any person treating a part of the human body like eye, the ear or the teeth, for a cure, is practicing a branch of medicine.

I am frank to say that any educational institution with the legal authority to confer a degree in any one of these branches should grant a degree having a medical tinge by ending with M. D. and the additional letter (in Latin,) indicating the branch or the specialty, thus making the dental degree D. M. D., and presenting the graduates with a degree indicating a dental medical education, an education so thoroughly tinged with medicine that there can be no question as to the graduate's ability to treat his patient systemically as well as locally for his cure.

This degree calls for a course of dental study so saturated with medicine that a graduate upon receiving it will be thoroughly qualified to treat any form of dental disease the mouth and teeth are heir to, without the aid of a physician. This does not mean that he should never consult with the physician. This course of study is to strengthen the union between the physician and the dentist in times of consultation.

We should make our course of study along these lines for our own name's sake and our future welfare.

What our country requires is better tooth doctors. I recommend this degree above all others, and the course of study for which the degree calls, to be adopted by all the schools as fast as they become able to present the course of study which the degree indicates, as the only possible way to procure an even, equally high, recognized standard of dental education in America. I say put us under one flag (D. M. D.,) and with the course of study which I have mentioned.

One of the reasons for which I recommend this degree is that M. D. covers everything in surgery. There is no surgical degree in medicine, and it is safe to say there never will be one. Therefore, I can see no good grounds for one in any of its branches.

Harvard stands to this country as does Oxford to England. Harvard selected this degree, and not without thought for the future. It was a wise and far-sighted selection. I I honor the institution for its choice and the course of study which the degree indicates. Harvard will never change this degree. She well knows it will do the same for her dental graduates as M. D. does for the medical graduates. Students educated along these lines and under one degree will retain their title of doctor, which has already been scratched in two countries. They will have retained the confidence of the mother profession (medicine) in times of consultation, and the entire profession for the cure of all material diseases will become united and "inseparable forever."—Dr. August L. Wells, Jr., Boston, Mass., in "Items of Interest."

---

TREATMENT OE ABSCESS SWELLING.—When I have a case of swelling resulting from the formation of abscess, I have no better treatment than the saline cathartic. I have never used a compound cathartic pill. It may be effective by producing a general depression, but the saline is the only remedy I know of for reducing a swelling after an abscess has opened.—Dr. Greenbaum, in "International."

OIL FOR APPENDICITIS.—Dr. M. O. Terry, surgeon-general of the National Guard of New York, adv cates the use of oleaginous cathartics in the treatment of appendicitis. He asserts that of fifty-one cases under his personal supervision, forty nine were successfully handled without operation. This treatmont ("Medical Times") is as follows: At first, cathartics of castor oil and sweet oil, followed by hot water, are given until the bowels are thoroughly cleaned out. This treatment is followed by enemas of glycerine and sweet oil. Flaxseed poultices soaked in sweet oil are kept on the abdomen. The diet is restricted to very light, easily-digested foods. The oil treatment, Dr. Terry says, removes the friction of the inflamed tissues and relaxes them during resolution. In this way, he says, he has cured cases of chronic recurrent appendicitis. To prevent a return of the trouble after the original treatment, he prescribes a tablespoonful of sweet oil followed by a glass of hot water, before each meal, for several weeks.—"Medical Brief."

---

EXTRACTION OF TEETH.—In general, the incisors and cuspids should be twisted from their sockets. The bicuspids rocked from side to side and twisted. The first and second molars are rocked and forced backward. The wisdom teeth should be tilted back with an elevator forced between it and the second molar and then taken from the socket with the forceps. The elevator well understood is the most useful of all extracting instruments, and the dentist who learns its use will relieve patients of stubborn roots that forceps will leave to worry them and cause maledictions to be heaped on the luckless tooth-puller.—Dental Hints.

---

TO REMOVE GUTTA-PERCHA POINTS FROM ROOT-CANALS.—Roughen the point of an Evans' root-canal dryer; heat the bulb and pass the point slowly into the canal. Cool the bulb with a wet sponge, and on removing the point the gutta-percha will come with it.—"Indiana Dental Journal."

A SCHOOL INSPECTION.—When Russia takes any matter in hand it is generally carried out in a thorough manner. I see that the Russian Public Health Society has appointed a committee, under the presidency of Dr. Limberg, for the organization of a regular system of dental inspection and treatment in schools, not only in St. Petersburg, but throughout the empire. As a preliminary step the committee has sent out a circular of questions to all heads of schools of the lower and middle grades, and to all practitioners having medical charge of such schools. It will be interesting, if these results are published, to compare them with the similar work which has been carried on for some years in some of our Board of Guardians' Schools, and by the lately-formed School Dentists' Society.—Dental Record.

---

A COUNTER-IRRITANT.—I instruct my patients to mix a little ginger, red pepper and mustard, and sprinkle a little on the fleshy part of a raisain, and then roast the raisen. This is an easy method of making a capital capsicum plaster in case of emergency. It acts like a charm generally, and is more effective than the roasted raisin by itself.—Dr. Roberts, International Dental Journal.

---

TO GIVE A FINE FINISH TO GOLD.—After the scratches have been removed with pumice stone, nothing is so effectual as oxide of zinc on a brush wheel. It leaves a beautiful, lustrous polish, and does not soil the hands.—H. H. Johnson, in "Dental Weekly."

